# Mixed-methods evaluation of a novel online STI results service

**DOI:** 10.1136/sextrans-2017-053318

**Published:** 2018-01-11

**Authors:** Jo Gibbs, Catherine R H Aicken, Lorna J Sutcliffe, Voula Gkatzidou, Laura J Tickle, Kate Hone, S Tariq Sadiq, Pam Sonnenberg, Claudia S Estcourt

**Affiliations:** 1 Centre for Population Research in Sexual Health and HIV, Institute of Global Health, University College London, London, UK; 2 Blizard Institute, Barts and The London School of Medicine and Dentistry, Queen Mary University of London, London, UK; 3 College of Engineering, Design and Physical Sciences, Brunel University London, Uxbridge, UK; 4 Applied Diagnostic Research and Evaluation Unit, Institute for Infection and Immunity, St George’s University of London, London, UK; 5 School of Health and Life Science, Glasgow Caledonian University, Glasgow, UK

**Keywords:** chlamydia infection, clinical sti care, testing, service delivery

## Abstract

**Objectives:**

Evidence on optimal methods for providing STI test results is lacking. We evaluated an online results service, developed as part of an eSexual Health Clinic (eSHC).

**Methods:**

We evaluated the online results service using a mixed-methods approach within large exploratory studies of the eSHC. Participants were chlamydia- positive and negative users of online postal self-sampling services in six National Chlamydia Screening Programme (NCSP) areas and chlamydia-positive patients from two genitourinary medicine (GUM) clinics between 21 July 2014 and 13 March 2015. Participants received a discreetly worded National Health Service ’NHS no-reply’ text message (SMS) informing them that their test results were ready and providing a weblink to a secure website. Participants logged in with their date of birth and mobile telephone or clinic number. Chlamydia-positive patients were offered online management. All interactions with the eSHC system were automatically logged and their timing recorded. Post-treatment, a service evaluation survey (n=152) and qualitative interviews (n=36) were conducted by telephone. Chlamydia-negative patients were offered a short online survey (n=274). Data were integrated.

**Results:**

92% (134/146) of NCSP chlamydia-positive patients, 82% (161/197) of GUM chlamydia-positive patients and 89% (1776/1997) of NCSP chlamydia-negative participants accessed test results within 7 days. 91% of chlamydia-positive patients were happy with the results service; 64% of those who had tested previously found the results service better or much better than previous experiences. 90% of chlamydia-negative survey participants agreed they would be happy to receive results this way in the future. Interviewees described accessing results with ease and appreciated the privacy and control the two-step process gave them.

**Conclusion:**

A discreet SMS to alert users/patients that results are available, followed by provision of results via a secure website, was highly acceptable, irrespective of test result and testing history. The eSHC results service afforded users privacy and control over when they viewed results without compromising access.

## Introduction

Timely provision of STI test results enables prompt treatment to reduce the risk of complications, prevent onward transmission and provide early opportunities for risk reduction. Notification of negative results gives reassurance and is an important opportunity for health promotion.

Services in the UK use a range of methods for communicating results, largely without good quality evidence,^1^ including: (1) face-to-face (patient returns to clinic), (2) telephone call, (3) letter, (4) automated telephone service, (5) short message service (SMS), (6) email and (7) online.

Efforts to improve efficiency have led many sexual health services to embrace eHealth for some elements of routine care, including results communication. We developed and piloted an eSexual Health Clinic (eSHC), which included an online chlamydia pathway (OCP) composed of an automated online clinical consultation for people with genital chlamydia, with electronic prescription via community pharmacy, partner notification and surveillance, supported by a telephone clinical helpline.^2^

The eSHC included a results service designed for confidential, private access to results and for chlamydia-positive users to continue onto the OCP. We evaluated it within large, mixed-methods exploratory studies of the eSHC.

## Methods

### Design of the online results service

Development of the eSHC^3^ included research to identify the optimal method for results provision. Two qualitative studies among 16–24 year old potential users explored the acceptability^4^ and usability^5^ of online STI care, including views and preferences about results provision. Findings suggested that avoiding referring to sexual health in electronic messages was important in maintaining privacy and that the National Health Service (NHS) ‘brand’ conferred legitimacy and trustworthiness.^4 5^

Informed by these findings and the literature,^1^ we opted for a two-step model. First, the eSHC sends users a discreetly worded SMS from the secure NHS SMS system stating that results are ready and providing a link to the eSHC web application. Second, users log on with their date of birth and mobile phone or clinic number to view results. For chlamydia-positive users, succinct information about the infection, links to relevant, reputable patient information websites and an offer of online management is provided (see figure 1 in the online Supplementary file 2). For users testing negative, information about the window period and health promotion advice are provided. The SMS and online text were cognitively tested to ensure ease of comprehension.^3^

10.1136/sextrans-2017-053318.supp2 Supplementary file 2

### eSHC evaluation

This results service was evaluated within proof-of-concept exploratory studies.^2^

Participants had undergone STI testing via: (1) an online postal self-sampling service (Checkurself) in six South London National Chlamydia Screening Programme (NCSP) areas and (2) two Greater London genitourinary (GUM) clinics between 21 July 2014 and 13 March 2015.^2^ GUM patients testing positive for chlamydia and NCSP participants testing positive or negative for chlamydia were eligible. Responsibility for notifying patients who had not accessed their results within 7 days was passed back to the relevant testing service, for those testing positive to be contacted by other means. Figure 2 in the online Supplementary file 1 illustrates how the results service was operationalised in different settings.

10.1136/sextrans-2017-053318.supp1 Supplementary file 1

### Methods evaluation of the online results service

The eSHC system automatically logged all interactions with the web application and their timing.

The acceptability of the results service was evaluated in: (1) a telephone survey of chlamydia-positive patients, administered in their clinical follow-up telephone call (2 weeks after test results were available); (2) qualitative interviews among a purposive sample of chlamydia-positive patients (20/36 female, aged 18–35) about using the eSHC,^6^ including results notification; and (3) an online survey of chlamydia-negative users.

Data were analysed descriptively. Free-text responses from survey questions and interview transcripts were used to explain and enrich the quantitative findings.

## Results

Ninety-two per cent (134/146) of chlamydia-positive NCSP patients, 82% (161/197) of chlamydia-positive GUM patients and 89% (1776/1997) of chlamydia-negative NCSP users accessed their results via the results service within 5 days. Of these, 97% of those testing positive (284/295) and 97% of those testing negative (1716/1776) accessed their results the day they received their text.

Sixty-nine per cent (152/221) of chlamydia-positive patients completed the telephone survey, and 36 qualitative interviews were conducted. Nineteen per cent (331/1776) of chlamydia-negative users completed the online survey.

### Use of the results service

Qualitative interviewees typically described accessing their results as soon as they noticed the SMS, irrespective of where they were. Those in public described being able to access their result with sufficient privacy from those around them, using their phones. For instance, one man, working in a shared office, described how ‘*on my mobile I was sure that nobody was looking*’ (24-year-old man). In the case of privacy concerns and constraints (eg, being particularly busy, lacking internet connectivity), some interviewees described accessing their results a short while later (‘*it’s not something I’d have wanted to open up on my desktop computer [at work]*’ (26-year-old man). They welcomed the online results service, for the ability it gave them to log on when they felt ready. Survey free-text responses reflected this concern for privacy, with some respondents commenting that they appreciated not having ‘chlamydia’ in the SMS.

### Acceptability

#### Chlamydia-positive patients

Of the 152 chlamydia-positive patients completing the telephone survey, 138 (91%) reported being happy with the online results service, although 7% (10/152) would have preferred their result to be displayed within the SMS (table 1).

**Table 1:** Acceptability of the STI results service: survey results

	Chlamydia-positive		Chlamydia-negative	
	Total (n)	Tested previously (n)	Tested previously (n)	Never tested before (n)
Thought the way they got results this time compared with previous experience/s was:				
Much better		14/41 (34%)	100/274 (36%)	
Better		13/41 (32%)	26/274 (17%)	
About the same		11/41 (27%)	97/274 (35%)	
Worse		3/41 (7%)	29/274 (11%)	
Much worse		0	0	
Would be happy to get results this way in the future			241/269 (90%)	
Happy with the way they got their results	138/152 (91%)			63/64 (98%)
If tested positive in the future would be happy to access results this way				61/64 (95%)
Would rather have got result via email with link to access result	0/152			3/62 (5%)
Would rather have result in text message	10/152 (7%)			27/62 (44%)
Text message was not clear	1/152 (1%)			
Amount of information given with results was:				
Not enough	1/108 (1%)			
About right	107/108 (99%)			
Too much	0/108			

Interviewees discussed the two-step process for accessing results positively, compared with receiving results in a message, ‘*cos you don’t know who’s gonna be like holding your phone*’ (26-year-old man). Logging on was generally described as straightforward.

Sixty-six per cent (27/41) of those who had tested for chlamydia previously reported preferring the online results service. However, some interviewees, familiar with receiving negative results directly in a text message, assumed that the message requiring that they log on, meant that they had tested positive. This affected feelings about logging on and the urgency of doing so. This woman described how she felt ‘*very apprehensive*’ and checked her result immediately: ‘*I wasn’t gonna wait[…] And I think it was a lot to do with the fact that it said “Your results are now ready to view online”. I’ve, I’ve never had anything before […] I just knew there was something, because usually it’d just be like ‘All of your results are negative*’ (22-year-old woman).

Ninety-nine per cent (107/108) of chlamydia-positive survey participants reported that the amount of information given with their online results was ‘about right’, which was typical of the qualitative interviewees.

#### Chlamydia-negative users

Ninety per cent (241/269) of the chlamydia-negative users with previous testing experience reported that they would be happy to use the results service again. Free-text responses indicated that some users appreciated the increased privacy and confidentiality, professionalism and security of the results service, despite the increased time it took to log on. However, others considered the log-on method insufficiently secure. Some chlamydia-negative users reported having made similar assumptions that being asked to log in meant their result was positive. Some users would prefer results by SMS, as this was faster and provoked less anxiety. Ninety-eight per cent (63/64) of those testing for the first time were happy with the online service but 44% (27/62) of these would have preferred their result displayed within the SMS. However, 53% (146/274) of chlamydia-negative participants who had tested previously reported that the online results service was better than their previous experience.

Twenty-eight per cent (389/1776) of chlamydia-negative users accessed the health promotion web page.

## Discussion

We developed and evaluated a novel two-stage process for online access to STI results underpinned by formative research. We have shown it to be an effective and acceptable way to provide positive and negative chlamydia test results. A high proportion of users accessed their results promptly, which is important from both an individual and public health perspective. The higher proportion of NCSP patients compared with GUM patients who accessed their results online is possibly because GUM patients may have expected to receive results by usual clinic practice.

We used different methods to evaluate acceptability in those testing positive and negative for chlamydia, which limited comparability, but allowed their differing experiences to be explored. The response rate for the online survey of chlamydia-negative patients was low (approximately 20%), but, arguably, the greater quality of research among those receiving positive results is appropriate given the epidemiological and clinical importance of ensuring effective communication of positive results, while maintaining privacy and security of sensitive patient data.

Despite the benefits to services of providing results via SMS,^7^ widespread use in clinics as the ‘default’ option, and uptake by patients, there is little evidence supporting its acceptability, particularly of displaying results in the message itself,^1^ and scant evaluation of alternative results communication methods. Many benefits of SMS results services, for example, mass provision of results in a cost-efficient/time-efficient manner, also apply to an online results service. In addition, an online results service allows users to access when and where it is convenient and can provide additional information to users. Previous studies found low acceptability of hypothetical online access to STI/HIV results,^8–10^ but since this research, both negative and positive HIV results have been provided online to men who have sex with men with high acceptability.^11^ Together with our results, this may indicate increased acceptability of online sexual healthcare over time.

Studies are underway to explore acceptability of providing positive test results online for STIs other than chlamydia, including HIV. As with the eSHC, these offer the potential for linkage to care and to risk-reduction interventions.
