# Supplementary figures and images for: Mixed-methods evaluation of a novel online STI results service
Source: Sex Transm Infect. 2018 Jan 11;94(8):622–4. doi: 10.1136/sextrans-2017-053318 (PMC6288705; doi:10.1136/sextrans-2017-053318)

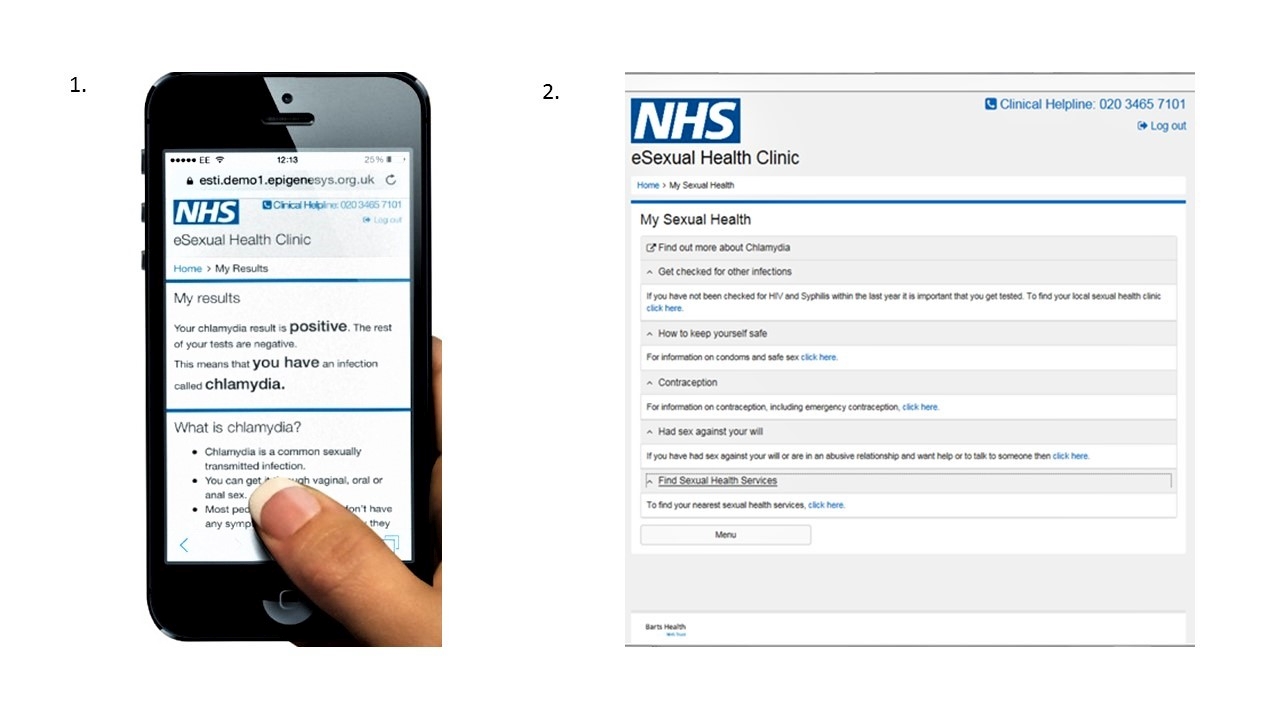

Supplement: Supplementary file 2 [file sextrans-2017-053318supp002.jpg]

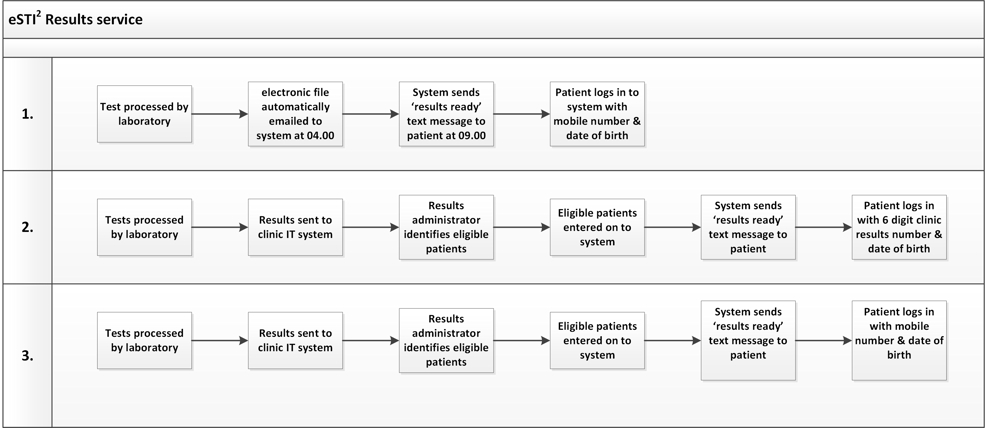

Supplement: Supplementary file 1 [file sextrans-2017-053318supp001.jpg]
